# Psychosocial Characteristics by Weight Loss and Engagement in a Digital Intervention Supporting Self-Management of Weight

**DOI:** 10.3390/ijerph18041712

**Published:** 2021-02-10

**Authors:** Ellen S. Mitchell, Qiuchen Yang, Heather Behr, Annabell Ho, Laura DeLuca, Christine N. May, Andreas Michaelides

**Affiliations:** 1 Academic Research, Noom, 229 W 28th St., New York, NY 10001, USA; qiuchen@noom.com (Q.Y.); hbehr@saybrook.edu (H.B.); annabell@noom.com (A.H.); ldeluca@mail.yu.edu (L.D.); christinem@noom.com (C.N.M.); andreas@noom.com (A.M.); 2 Department of Integrative Health, Saybrook University, 55 W Eureka St, Pasadena, CA 91103, USA; 3 Ferkauf Graduate School of Psychology, Yeshiva University, 1165 Morris Park Ave, Bronx, NY 10461, USA

**Keywords:** self-managed digital program, digital health, demographics, weight loss

## Abstract

There is substantial variability in weight loss outcomes. Psychosocial characteristics underlying outcomes require better understanding, particularly on self-managed digital programs. This cross-sectional study examines differences in psychosocial characteristics by weight loss and engagement outcome, and which characteristics are most associated with weight loss, on a self-managed digital weight loss program. Some underexplored psychosocial characteristics are included, such as flourishing, or a sense of meaning and purpose in life. A questionnaire was emailed to a random sample of 10,000 current users at week 5 in the program and 10,000 current users at week 17. The questionnaire was completed by 2225 users, and their self-reported weight and recorded program engagement data were extracted from the program’s database. Multiple comparison tests indicated that mental health quality of life, depression, anxiety, work-life balance, and flourishing differed by weight loss outcome at program end (week 17; ≥5%, 2–5%, below 2%) and by engagement tertile at program beginning and end (weeks 5 and 17). Only anxiety was associated with weight loss in a backward stepwise regression controlling for engagement and sociodemographic characteristics. Flourishing did not predict weight loss overall but predicted the weight loss outcome group. Our findings have implications for creating more effective interventions for individuals based on psychosocial characteristics and highlight the potential importance of anxiety in underexplored self-managed digital programs.

## 1. Introduction

Obesity impacts 37.7% of adults in the US and is associated with an increased risk of major health conditions such as hypertension and diabetes [1,2]. One of the most effective treatments is behavioral lifestyle modification that results in modest weight loss [3]. However, there is substantial variability in weight loss outcomes and engagement with program behaviors, an important indicator of weight loss success [4,5,6]. To help explain this variability, considerable attention has been devoted to individuals’ psychosocial characteristics [7,8], which could help to tailor treatments to individuals, identify individuals that might be at risk for suboptimal outcomes, and ensure more optimal outcomes and behaviors [7,8]. Additionally, there is even less understanding of the role of psychosocial characteristics for digital lifestyle modification interventions, where individuals must manage all their behaviors in the absence of in-person sessions and recruitment. Most individuals may attempt to lose weight in a self-managed program or on their own, rather than in clinical settings [4,9]. Little research has explored psychosocial characteristics associated with weight loss and engagement in this context, though they may manifest differently than what has been demonstrated in clinical studies [4,10].

Studies of weight loss trials, primarily in clinical settings, report mixed findings on the psychosocial characteristics that relate to weight loss and engagement. In terms of weight loss, a recent meta-analysis found that very few psychosocial characteristics showed a consistent relationship with weight loss [8]. For example, some studies find a significant association between weight loss and characteristics such as sleep quality, depression, anxiety, and mental health quality of life [8,11,12,13,14], while others do not [8,13,15,16,17]. Therefore, knowledge in this area is still limited, and more work is needed on a broader range of characteristics [8,18,19]. For example, to date, no study has explored the relationship between work-life balance and weight loss, even though having a poorer balance between work and life is linked to lower amounts of physical activity and pronounced stress-induced eating [20].

More understanding is also needed of psychosocial characteristics related to engagement. Previous studies have focused primarily on attrition and adherence, or the extent to which individuals follow program recommendations [6]. Very few studies have examined psychosocial characteristics related to engagement, a key indicator of success on a digital intervention. Engagement has been defined as the amount of relevant behavior indicative of motivating behavior change necessary for improved outcomes [5]. Some studies have found associations between adherence or attrition and psychosocial characteristics such as depression, anxiety, and mental health quality of life [6,21,22,23,24,25], with some studies on anxiety finding no significant associations [24,26]. One study on engagement specifically found that depression and anxiety did not significantly predict engagement in a digital mental health intervention [27]. Limited evidence has been found for sleep quality and work-life balance, though work-life balance was associated with non-completion of a wellness intervention [28].

Additionally, so far, there has been only limited work investigating the role of a potentially important psychosocial characteristic—flourishing—in weight loss. Well-being is thought to be composed of two aspects: hedonic, or emotion, and eudaimonic, or subjective perception of one’s life [29,30]. Flourishing has been conceptualized as eudaimonic well-being and constitutes the sense that one’s life has meaning, purpose, and connectedness [30]. Flourishing is linked to health outcomes and behaviors. For instance, lower levels of flourishing are associated with higher mortality rates even after adjusting for important covariates, as well as lower exercise, higher alcohol consumption, and greater restless sleep [31,32]. Higher flourishing is also associated with a greater intake of fruit and vegetables [33]. In cross-sectional surveys, individuals with class 2 or class 3 obesity reported significantly lower levels of flourishing than individuals with overweight or normal weight, adolescents with obesity had significantly lower levels of flourishing than adolescents with normal weight, and weight loss maintainers reported marginally significantly higher flourishing levels than non-maintainers [19,34,35]. In the latter study, flourishing and related factors accounted for 24% of the variance in BMI, highlighting the potential utility of exploring flourishing, particularly given current insufficient understanding of psychosocial characteristics. However, no study has yet explored the associations between sociodemographic factors and flourishing in a weight loss program.

Therefore, this study’s aim is to broaden the scope of psychosocial characteristics examined, including flourishing and work-life balance; examine which characteristics distinguish individuals with higher levels of weight loss and engagement from those with moderate and lower levels; and provide an ecologically valid view of these outcomes as they naturally occur without additional guidance from researchers on a self-managed digital weight loss program. In a cross-sectional survey of current users of a self-managed digital weight loss program, we first explored the nature of flourishing, with the expectation that education, marital status, gender, and general health will be associated with flourishing, but without specific hypotheses for the psychosocial characteristics and remaining sociodemographic characteristics due to limited past research [36]. We hypothesized given the patterns demonstrated in past research that self-reported mental health quality of life, depression, anxiety, work/life balance, sleep habits, and flourishing at program end would differ between individuals with meaningful, moderate, and low weight loss. Based on prior work on attrition and adherence, we hypothesized that mental health quality of life, depression, anxiety, sleep habits, work/life balance, and flourishing would significantly differ between individuals with meaningful, moderate, and low engagement. We next explored which of these psychosocial characteristics at program end was most associated with weight loss, when accounting for sociodemographics and engagement. This investigation may help to provide a practical understanding that can help to create more effective programs and provide insight into the many individuals who self-manage their weight loss on digital platforms outside of formal trials.

## 2. Materials and Methods

Mixed prior results highlight the need for a better understanding of how psychosocial characteristics impact weight loss, which this study aims to contribute in several ways. First, we included a large sample of individuals to maximize sample size, as recommended by reviews such as [4,7]. Second, instead of focusing on the predictive value of baseline characteristics, we explored which psychosocial characteristics distinguish high weight loss and engagement from moderate and lower amounts. This type of analysis lends itself to better practical understanding, such as how to tailor programs to specific individuals or what psychosocial benefits occur other than weight loss [7]. We do this within the underexplored context of a self-managed digital weight loss program. Third, we explore a broader range of psychosocial characteristics, particularly those that are potentially important but remain underexplored [8,21], such as flourishing.

### 2.1. Program

Noom is a mobile health behavior change weight loss program that has demonstrated effects on clinically significant weight loss [37,38]. The Noom Healthy Weight program is based on Cognitive Behavioral Therapy (CBT), third-wave CBT, and motivational interviewing techniques, all of which aid in behavior change and weight control [39,40,41,42]. Users self-enroll in the program by purchasing it from the app store (iTunes/Google Play) or the website. Users are provided access to a curriculum; food, exercise, and weight logging features; and a virtual 1 on 1 coach and group, which all have been demonstrated to be effective components of weight-loss interventions [43,44]. The curriculum involves daily articles on psychological principles, diet, physical activity, weight management, and behavior change. Coaches, trained to use techniques from cognitive behavioral therapy and motivational interviewing, interact with users to help them set health goals and to support them based on their progress and needs [45]. Users are encouraged to log their weight daily.

### 2.2. Participants

Participants included adults who voluntarily signed up for Noom Healthy Weight in either the month of September 2020 or June 2020, and were therefore in their 5th or 17th week at the time of data collection. These time points were chosen based on the length of the core program (16 weeks), in which the 17th week constitutes core program completion and the 5th week constitutes the first post-trial week. We used the first week past the free trial to ensure that all participants had chosen to self-manage their weight loss on the full program. All users provided informed consent to participate in research during sign-up and were given the option to opt-out, and the study received IRB approval. Of users who did at least one in-app action per month (e.g., inputted a body weight measurement), a random sample of 10,000 users in their 5th week and a random sample of 10,000 users in their 17th week were emailed a questionnaire invitation and link and were not compensated for their participation. There were 2571 users who responded to the questionnaire. Participants’ weight, which is self-reported in the program, was de-identified and extracted from Noom’s database. De-identified engagement data were both self-reported (meals, exercises, weigh-ins) and objectively measured (steps, articles read, messages sent to the coach) and extracted from Noom’s database. Survey responses were matched to participants’ weight and engagement data by email address and de-identified before analysis. Participants who could not be matched based on email addresses were excluded from analyses. Participants were also excluded if they did not provide their self-reported weight at baseline. Following past work, outliers of BMI changes of more than 3.5 within a month were excluded [46]. One outlier in terms of baseline weight (self-reported as 20 kg) was removed. A total of 2225 participants were included in analyses, with 1133 in week 5 and 1092 in week 17.

### 2.3. Measures

Primary measures were weight loss and engagement. Weight loss was calculated using participants’ self-reported weight at baseline subtracted from self-reported weight at the end of week 16, only for participants at week 17. Engagement was assessed for both groups using the following measures of weight loss behaviors recorded by the program, which have been found to significantly predict weight change: total number of self-reported meals and exercises, recorded steps, messages sent to the coach, articles read, and days with one weigh-in each week. These variables were averaged over the number of weeks in the program, then converted to z-scores. The six z-scores were summed to create an overall engagement score for each participant.

Participants were asked to complete a questionnaire assessing sociodemographic factors and psychosocial characteristics at the time of data collection, which constituted either week 5 or week 17 in the program.

#### 2.3.1. Sociodemographic Measures

Participants were asked their race, ethnicity, general health status, employment status, marital status, education level, gender, household income, diagnosed health conditions, the number of medications taken, COVID status (i.e., social distancing, self-isolating, under a shelter or stay at home order, hospitalized, or diagnosed with COVID-19), and the number of children living with the participant. In addition, baseline body mass index (BMI) was calculated using users’ self-reported weight and height at baseline.

#### 2.3.2. Psychosocial Measures

Measures were chosen based on previous literature on psychosocial characteristics, particularly those that might be important in a digital behavior change weight loss program but have been underexplored in previous work. Participants were asked about their mental health quality of life, depression, and anxiety via three items from the Quality of Life subsection of the Center for Disease Control and Prevention (CDC)’s Behavioral Risk Factor Surveillance System (BRFSS) survey [47]. The BRFSS survey is an annual federal survey developed by the CDC. The HRQOL-14 is a subscale of the BRFSS that assesses mental health quality of life, depression, and anxiety, and has acceptable validity and psychometric properties [48,49]. For mental health quality of life, users were asked how often they “felt stressed, depressed, or had problems dealing with your emotions” over the past 30 days, with a response scale ranging from 1 (not at all) to 5 (extremely often). Because the mental health quality of life item assesses amounts of stress and difficulty handling emotions, lower scores indicate higher mental health quality of life. No cut-offs were used and all responses were used for analysis. For depression, users were asked how many days they felt “sad, blue, or depressed” over the past 30 days, with possible responses of “not at all”, “occasionally”, “more than half the days”, and “nearly every day”. For anxiety, participants were asked how many days they felt “worried, tense, or anxious” over the past 30 days, with response options “not at all”, “occasionally”, “more than half the days”, and “nearly every day”. As with mental health quality of life, no cut-off scores were used and all responses were included in the analysis. Work-life balance was assessed with an item adapted from [50] and revised to match the time period of the BRFSS questions (over the past 30 days). An overall perceived sleep quality question from [51] was adapted to assess overall perceived sleep habits. Finally, the 8-item Flourishing Scale [30] was used to measure flourishing.

#### 2.3.3. Statistical Analyses

Weight loss groups were defined as meaningful weight loss (at least 5%), moderate weight loss (2–5%), and low weight loss (below 2%), based on CDC standards for clinically meaningful weight loss (5%) and prior evidence for beneficial health effects with weight loss starting at 2% [52]. Engagement groups were delineated into tertiles based on distributions of z-scores. Descriptive statistics are expressed in means and standard deviations, as well as percentages. First, a stepwise regression model was used to explore the most prominent associations between sociodemographic and psychosocial variables and flourishing. Second, to assess whether psychosocial characteristics differed across the three weight loss and engagement groups, ANOVAs were used for continuous variables, and chi-square tests were used for categorical variables. Next, univariate regressions were conducted to examine which of those psychosocial characteristics are associated with weight loss and engagement. Each psychosocial variable was the independent variable in its own separate regression with weight loss or engagement as the dependent variable. A backward stepwise regression model with all characteristics as dependent variables and weight loss as the independent variable was then conducted to understand which psychosocial characteristics were most associated with weight loss while accounting for sociodemographic characteristics and engagement. The least informative covariates were successively removed from the model in a backward stepwise elimination procedure based on the Akaike information criterion (AIC). Only week 17 responses were used for any analysis involving weight loss since full weight loss from the program is not yet evident for week 5 participants. Week 5 and 17 responses were combined for all analyses involving engagement for two reasons. First, week 17 sociodemographic data did not substantially differ from week 5 data. Second, using the combined data provides a more accurate view of engagement, since program engagement is thought to decrease over the course of a program because of successful adoption of health behaviors [5]; therefore, lower engagement at week 17 would not necessarily mean low engagement with weight loss behaviors. All analyses were carried out in R (version 3.6.0).

## 3. Results

### 3.1. Sociodemographic Characteristics

Demographic characteristics for the entire sample are displayed in Table 1. The sample was mostly White (87.2%, N = 1941), non-Hispanic or Latino (93.8%, N = 2088), married (67.6%, N = 1504), and female (83%, N = 1847). 53.5% (N = 1190) reported being employed full-time. 71.6% (N = 1592) indicated having a 4-year degree or higher, 60% (N = 1335) having a health condition, 63% (N = 1401) taking medication, 81% having good or very good self-reported health, and 96.5% (N = 2148) social distancing during the past four weeks.

### 3.2. Sociodemographic and Psychosocial Characteristics Associated with Flourishing

All demographic characteristics as well as psychosocial characteristics were entered into a stepwise regression model to assess which ones were most associated with flourishing. As expected, being able to work (i.e., employed, looking for work, retired, or not employed and not looking for work) was associated with significantly higher flourishing compared to being disabled and unable to work (B = 3.01, *p* = 0.005; B = 3.32, *p* = 0.002; B = 3.45, *p* = 0.004; B = 2.39, *p* = 0.04; B = 3.94, *p* < 0.001). Being male was associated with lower flourishing (B = −0.82, *p* = 0.008). Having good or very good health compared to fair health was associated with higher flourishing (B = 2.71, *p* <0. 001; B = 4.54, *p* < 0.001), while having poor health was associated with lower flourishing (B = −2.57, *p* = 0.01). In terms of education, only having a graduate degree was associated with higher flourishing compared to having a 2-year college degree (B = 0.86, *p* = 0.04). Being married or married with children were also associated with higher flourishing compared to being divorced (B = 1.16, *p* = 0.009; B = 1.49, *p* < 0.001). The psychosocial variables that emerged in the stepwise model were mental health quality of life (B = −0.35, *p* = 0.003), depression (nearly every day: B = −4.86, *p* < 0.001; not at all: B = 5.34, *p* < 0.001; occasionally: B = 3.23, *p* < 0.001), work-life balance (B = 0.55, *p* < 0.001), and sleep quality (B = 0.43, *p* < 0.001), but not anxiety. Therefore, the characteristics expected to relate to flourishing showed significant associations with flourishing.

### 3.3. Psychosocial Characteristics by Weight Loss Outcome

Psychosocial characteristics were examined by weight loss outcome group (see Table 2). Mental health quality of life (*F*(2,1022) = 11.49, *p* < 0.001), depression (*X2*(6, *n* = 1025) = 26.84, *p* < 0.001), anxiety (*X2*(6, *n* = 1025) = 26.09, *p* < 0.001), and work-life balance (*F*(2,1022) = 4.77, *p* = 0.009) significantly differed across weight loss outcome groups. Flourishing also significantly differed across groups (*F*(2,1022) = 3.44, *p* = 0.03). These psychosocial variables showed linear patterns across the three groups. For example, individuals in the low weight loss group had the highest depression, followed by the moderate weight loss group, with individuals in the high weight loss group showing the lowest levels of depression. Similar linear patterns emerged for mental health quality of life (reverse-scored), anxiety, work-life balance, and flourishing. Self-reported sleep habits did not significantly differ across groups.

Post-hoc pairwise comparison tests were conducted to identify which of the three weight loss groups were significantly different from each other in psychosocial characteristics. Significance values were adjusted for multiple comparisons using the Holm-Bonferroni method [53]. The meaningful weight loss group had significantly lower depression and anxiety, as well as higher mental health quality of life and work-life balance than the low weight loss group. These two groups did not significantly differ in terms of flourishing after the Holm-Bonferroni adjustment. Only mental health quality of life significantly differed between the meaningful and moderate weight loss groups. Other characteristics such as anxiety and flourishing approached but did not reach statistical significance. None of the differences between the moderate and low weight loss groups approached statistical significance. Therefore, the biggest differences in psychosocial characteristics emerged between the meaningful and low weight loss groups.

### 3.4. Associations with Weight Loss

Univariate analyses were conducted to understand which of these psychosocial characteristics may be related to weight loss. Univariate regressions indicated that of these psychosocial characteristics, mental health quality of life (B = −0.60, *p* < 0.001), being “not at all” depressed compared to “more than half the days” (B = 1.68, *p* = 0.003), and being “not at all” anxious or “occasionally” anxious compared to “more than half the days” (B = 1.53, *p* = 0.003; B = 1.60, *p* < 0.001) at week 17 were significantly associated with weight loss at week 17. Work-life balance (B = 0.11, *p* = 0.11) and sleep habits (B = 0.12, *p* = 0.10), were not associated with weight loss at week 17; neither was flourishing (B = 0.02, *p* = 0.40). This suggests that of the psychosocial characteristics that distinguished weight loss groups from each other at week 17, only mental health quality of life, depression, and anxiety were associated with weight loss. Higher levels of mental health quality of life, depression, and anxiety were associated with greater weight loss.

Therefore, flourishing was not associated with weight loss overall despite significant differences in flourishing among weight loss groups and expected associations between sociodemographics and flourishing. To better understand this discrepancy, we conducted a post-hoc analysis. Given the majority of users were in the meaningful weight loss group (N = 618 out of 1025), we speculated that the discrepancy could have arisen if flourishing does not predict weight loss in larger amounts but predicts weight loss up to a certain point. Therefore, we conducted a post-hoc ordinal logistic regression with the weight loss outcome group (meaningful, moderate, low) as the dependent variable and flourishing as the independent variable. Flourishing was a significant predictor of the weight loss outcome group (B = −0.02, *p* = 0.01). For every one-unit increase in flourishing, the odds of being in the meaningful or moderate weight loss groups increases by 2.6%.

### 3.5. Psychosocial Characteristics by Engagement Outcome

Psychosocial characteristics were then compared across tertiles of engagement, another outcome of interest (see Table 3). The tertile with the highest engagement had z-scores greater than or equal to 1.68 for week 5 participants or 1.93 for week 17 participants. The tertile with medium engagement had z-scores between −1.06 and 1.67 for week 5 participants or −1.31 and 1.92 for week 17 participants. The tertile with the lowest engagement had z-scores less than −1.06 for week 5 participants or −1.31 for week 17 participants.

Mental health quality of life (*F*(2,2055) = 9.71, *p* < 0.001), depression (X2(6) = 26.84, *p* < 0.001), anxiety (X2(6) = 26.54, *p* < 0.001), work-life balance (*F*(2,2055) = 9.71, *p* < 0.001), and sleep habits (*F*(2,2055) = 10.31, *p* < 0.001) significantly differed across engagement tertiles, and showed linear patterns across the three groups, with the highest mental health quality of life, depression, anxiety, work-life balance, and sleep habits in the high engagement, followed by the moderate engagement and low engagement tertiles. Similarly, flourishing significantly differed across engagement tertiles (*F*(2,2055) = 11.01, *p* < 0.001).

Post-hoc pairwise comparison tests were used to ascertain which of the three engagement tertiles differed from each other on psychosocial characteristics. After Holm-Bonferroni’s adjustment for multiple comparisons, the high engagement tertile differed from the low engagement tertile on all psychosocial characteristics. The high engagement tertile had more optimal mental health quality of life, depression, anxiety, work-life balance, sleep habits, and flourishing compared to the low engagement tertile. The moderate engagement tertile had more optimal mental health quality of life, depression, anxiety, and work-life balance compared to the low engagement tertile, with no significant differences in sleep habits and flourishing after Holm-Bonferroni correction. Only anxiety significantly differed between the high and moderate engagement tertiles. This suggests that the biggest differences lay between the high and low engagement tertiles, followed by the moderate vs. low engagement tertiles.

### 3.6. Associations with Engagement

Of the psychosocial characteristics that distinguished engagement groups from each other, only anxiety was significantly associated with engagement in univariate regressions. Feeling “not at all” worried, tense, or anxious was significantly more associated with engagement (B = −1.07, *p* = 0.03) compared to the reference value of “more than half the days”, with no other differences for the other amounts of anxiety. In other words, feeling no anxiety yielded more engagement at week 17. No other significant associations emerged. Flourishing also was not associated with engagement (B = −4.52, *p* = 0.84).

### 3.7. Most Important Predictors of Weight Loss

All psychosocial characteristics with *p* < *0*.10 in univariate analyses were entered into a backward stepwise elimination multiple regression model to determine the most impactful factors for weight loss, accounting for sociodemographic factors and engagement. The final model had an R2 of 0.31 and contained 5 variables: living with children, gender, anxiety, engagement, and baseline BMI (see Table 4). Living with 1–2 children (compared to no children; B = 2.84, *p* < 0.001), being male (B = 3.31, *p* < 0.001), and being “occasionally” anxious compared to “more than half the days” (B = 0.94, *p* = 0.004) were associated with greater weight loss. Being “not at all” anxious was a marginally significant predictor of weight loss (B = 0.83, *p* = 0.06). Having low or medium engagement compared to high engagement (B = −4.75, *p* <.001; B = −1.45, *p* < 0.001) was associated with less weight loss. Having a higher initial BMI (B = 0.11, *p* < 0.001) was associated with greater weight loss, which is a pattern that has been found in other studies but could also signal regression to the mean in which participants with the largest differences from the mean lost the most weight [8,54]. This suggests that of the psychosocial characteristics considered, anxiety (and specifically being “occasionally” anxious compared to “more than half the days”) was most associated with the amount of weight lost when accounting for engagement and sociodemographic variables.

## 4. Discussion

This study is one of the first studies to examine psychosocial characteristics related to weight loss and engagement in the context of a self-managed digital weight loss program, and thus addresses gaps in existing understanding in several ways. First, there is limited understanding of relationships between weight loss, engagement, and individual-level characteristics on self-managed weight loss programs, despite the many individuals who use them [4]. Second, the study adds practical understanding by exploring which psychosocial characteristics distinguish weight loss and engagement outcomes from each other, which can inform efforts to personalize programs for better effectiveness and engagement [21]. Third, it broadens the scope of psychosocial characteristics by examining flourishing, which has been rarely studied in the context of weight loss [19]. In a survey of users participating in the Noom digital behavior change weight loss program, we found that mental health quality of life, depression, anxiety, work-life balance, and flourishing at week 17 differed across meaningful weight loss (5% and above), moderate weight loss (2–5%), and low weight loss (below 2%) groups. Mental health quality of life, depression, anxiety, flourishing, work-life balance, and sleep habits differed between high, medium, and low engagement groups. Of these psychosocial characteristics, only anxiety was associated with weight loss when accounting for sociodemographic characteristics and engagement.

Anxiety has been linked to obesity because it is associated with binge eating and unrestrained eating behaviors, and could result in decreased physical activity due to social avoidance [14,55,56]. We found that the frequency of perceived worry, tenseness, or anxiety over the past 30 days had the strongest association to weight loss out of all psychosocial variables examined. This could be an important psychosocial factor to consider in future studies using digital interventions. There are limited studies that have investigated whether anxiety or worry is associated with weight loss in a weight loss intervention, and null results have been found when anxiety is conceptualized as an individual’s current clinical level of anxiety. Using diagnostic interviews, one study found that changes in clinical anxiety did not differ by intervention condition [17], and having an anxiety disorder was not associated with weight loss in a conventional weight loss treatment program in [14]. However, a baseline composite of stress, anxiety, and depression, as measured by the General Health Questionnaire, significantly predicted weight loss at 6 months in [16]. This, along with our results, suggest that lower thresholds of anxiety should be considered in future studies, particularly for populations that are not expected to have a substantial prevalence of clinically diagnosed anxiety disorders. Our results also highlight the unique explanatory role of anxiety when it is conceptualized as the *frequency* of worry or anxiety. Future work should examine if there are substantial differences between framing anxiety in terms of frequency or as individuals’ current level at the moment.

We found that experiencing “occasional” anxiety compared to experiencing anxiety “most of the days” is most predictive of weight loss. Experiencing no anxiety compared to “most of the days” was marginally significant but did not reach significance. This suggests that there could be an amount of anxiety that is optimal for success in weight loss. Aligning with these findings, studies in other domains have found a curvilinear relationship in which performance is highest for some level of anxiety but not too much [57,58]. A slightly different pattern emerged for engagement in which having no anxiety was most predictive of active engagement behaviors on the program. Future research should explore to what extent there is some moderate level of anxiety that is optimal for weight loss success, and how that differs from optimal anxiety levels for active engagement. For example, studies can explore optimal anxiety levels for motivation to engage in these behaviors. Overall, both sets of findings suggest that lower levels of anxiety are more effective than higher levels of anxiety (e.g., “more than half the days” or “nearly every day”). This suggests that interventions should focus on decreasing the highest levels of anxiety, since this level of anxiety may be detrimental to both engagement and weight loss success.

Our results suggest that anxiety plays a greater role in weight loss than flourishing, but that flourishing plays a role up to a certain point. First, we found that expected characteristics were most associated with flourishing, such as education, marital status, gender, general health, mental health quality of life, depression, work-life balance, and sleep habits [36,59,60]. Flourishing significantly differed across weight loss groups and engagement tertiles. However, flourishing was not significantly associated with weight loss in univariate and stepwise regressions. In addition, more than half of the data came from the meaningful weight loss group (at least 5%), raising the possibility that flourishing predicts weight loss up to 5%, at which extensive variability (e.g., large weight loss amounts) is best predicted by other factors. Providing further support for this notion, in a post-hoc ordinal logistic regression predicting weight loss outcome group (meaningful, moderate, low), flourishing was significantly associated with weight loss (B = 0.02, *p* < 0.01). A one-unit increase in flourishing was associated with a 2.6% increase in odds of losing meaningful or moderate amounts of weight. This suggests that perhaps flourishing best predicts variability in low up to meaningful weight loss outcomes, but perhaps not beyond clinically meaningful (5%) levels. Future studies should further examine the role of flourishing in weight loss, particularly with prospective measurements.

This is one of the first studies to examine psychosocial characteristics related to engagement on a digital weight loss program, which is an important predictor of weight loss and health behaviors [5]. Prior studies have mostly examined only demographic characteristics or focused on adherence and attrition as opposed to engagement [12,61,62,63]. For example, one study of a digital gestational weight control program investigated the likelihood for different engagement groups to be in certain demographic and BMI subgroups [63]. Our results point to several psychosocial characteristics that distinguished engagement levels from each other. Notably, we found that sleep habits differed across engagement groups, though not across weight loss groups. Future research should determine if this is because sleep habits play a role in engagement or whether it is because more engaged people perceive themselves to have better sleep habits, especially with the use of objective measures of sleep. To our knowledge, this is the first study to demonstrate that levels of flourishing, depression, and anxiety differed among engagement levels, highlighting the need for additional research on the relationship between these factors and engagement.

While the majority of users in this sample (60%) were diagnosed with a health condition, there was a lower prevalence of diabetes (4.8%) and hypertension (18.7%) than reported prevalence in the general US adult population with overweight or obesity (diabetes: 13%, hypertension: 29%) [64,65]. This could be because the Noom Healthy Weight program is a generalized program for behavior change surrounding weight control for overweight and obesity, and users with needs that are more distinct to diabetes or hypertension may prefer diabetes or hypertension-specific programs. Future studies should explore whether the psychosocial characteristics explored in this study generalize to populations with higher incidences of diabetes and hypertension.

There are several limitations to this study. Despite providing an ecologically valid view of naturally occurring weight loss and behaviors, the cross-sectional, retrospective design does not make it possible to infer causal relationships. In addition, only 2571 users responded to the questionnaire, which could mean that sampling bias is possible if the psychosocial and demographic characteristics found in this population manifest differently in the full population of users. The scope of this study did not include predicting future outcomes using baseline characteristics but focused on characteristics distinguishing weight loss outcomes at the end of the program and engagement outcomes at both beginning and end. Therefore, for this particular study, it does not matter whether these psychosocial characteristics were affected by the program or were already existing at those levels in individuals from the start. Future studies, however, should incorporate baseline measures to explore the effects of the program and existing levels of psychosocial characteristics. The size of the questionnaire was also limited by constraints and could not include lengthy validated scales for every psychosocial factor or assess clinical nature, but future research should extend our findings with additional psychometrically valid scales.

## 5. Conclusions

There has been much work exploring correlations between weight or BMI and psychosocial characteristics in a variety of populations (e.g., [35,66,67,68,69]. However, more understanding is needed of individuals’ psychosocial characteristics as they control their weight and engage with digital interventions or programs. There is a particular paucity of research on digital programs that support self-management. Therefore, in a cross-sectional survey, we assessed psychosocial characteristics at various outcome levels in users of Noom, a digital behavior change weight loss program. Self-reported mental health quality of life, depression, anxiety, work-life balance, and flourishing significantly differed depending on weight loss outcome (5%, 2–5%, less than 2%). The same psychosocial characteristics with the addition of sleep habits significantly differed depending on engagement outcome. Flourishing significantly predicted the weight loss outcome category and was associated with expected sociodemographic characteristics. When all psychosocial characteristics were included in a stepwise regression controlling for engagement and sociodemographic characteristics, anxiety was the strongest predictor of weight loss. Our results suggest that flourishing, as well as mental health quality of life, depression, and work-life balance, may be influential in outcomes up to a certain extent. Our results also highlight the important influence of anxiety in weight loss in this population. Future research should investigate the nature of this anxiety, exploring whether it is generalized anxiety or related specifically to weight management.

Our results indicate that regardless of any program improvements on these psychosocial variables, there was still significant variation on these psychosocial characteristics by week 17. Therefore, while our correlational results do not allow for causal interpretations, they provide the first step towards honing in on psychosocial characteristics to focus on in future experimental studies. Implications for researchers and practitioners include two possibilities, depending on the direction of causality. First, if individuals’ psychosocial characteristics, whether inherent or shaped by the program, influenced weight loss and engagement outcomes, future programs can measure and attend to individuals’ mental health quality of life, depression, and in particular, anxiety for more effective weight loss and engagement. For instance, programs can individually tailor based on certain psychosocial characteristics to match the levels seen with optimal outcomes at the desired time-points. Future studies should experimentally assess the impact of targeting these psychosocial characteristics compared to the normal implementation of the program. Second, if the psychosocial outcomes were primarily influenced by weight loss outcomes, then programs and future studies can consider mental health quality of life, depression, and anxiety as indirect program benefits in themselves. Regardless of the direction of causality, our results suggest that mental health quality of life, depression, and especially anxiety should be further studied.

## Figures and Tables

**Table 1:** Overall sociodemographic characteristics for all respondents (*n* = 2225).

	N (%) or Mean (SD)
Hispanic	
No, not Hispanic/Latino	2088 (93.8%)
Yes, Cuban	8 (0.4%)
Yes, Mexican, Mexican American, Chicano	49 (2.2%)
Yes, Puerto Rican	15 (0.7%)
Yes, other Spanish/Latino	47 (2.1%)
Prefer not to answer	18 (0.8%)
Race	
American Indian or Alaska Native	15 (0.7%)
Asian	62 (2.8%)
Black or African American	72 (3.2%)
Native Hawaiian or other Pacific Islander	5 (0.2%)
White	1941 (87.2%)
Two or more races	75 (3.4%)
Prefer not to answer	55 (2.5%)
Employment status	
Disabled, not able to work	26 (1.2%)
Employed, working 1–39 h per week	512 (23.0%)
Employed, working 40 or more hours per week	1190 (53.5%)
Not employed, looking for work	78 (3.5%)
Not employed, NOT looking for work	105 (4.7%)
Retired	314 (14.1%)
Marital status	
Divorced	176 (7.9%)
In a relationship	176 (7.9%)
Married	736 (33.1%)
Married with children	768 (34.5%)
Single	317 (14.2%)
Widowed	52 (2.3%)
Do you have children living with you?	
No	1204 (54.1%)
Yes, 1–2 kids	831 (37.3%)
Yes, 3–4 kids	180 (8.1%)
Yes, 5+ kids	10 (0.4%)
What is your highest level of education?	
graduate degree (Master’s, PhD, MD, JD)	754 (33.9%)
4-year college degree	838 (37.7%)
2-year college degree	193 (8.7%)
Some college	281 (12.6%)
High school degree	93 (4.2%)
Some high school	10 (0.4%)
Vocational training	51 (2.3%)
I prefer not to answer	5 (0.2%)
How much total combined money did all members of your household earn in 2019?	
$0–9999	15 (0.7%)
$10,000–19,999	36 (1.6%)
$20,000–29,999	54 (2.4%)
$30,000–39,999	72 (3.2%)
$40,000–49,999	90 (4.0%)
$50,000–59,999	126 (5.7%)
$60,000–69,999	109 (4.9%)
$70,000–79,999	132 (5.9%)
$80,000–89,999	132 (5.9%)
$90,000–99,999	138 (6.2%)
$100,000 or more	989 (44.4%)
Prefer not to answer	331 (14.9%)
NA	1 (0.0%)
Gender	
Female	1847 (83.0%)
Male	366 (16.4%)
Other	2 (0.1%)
Prefer not to answer	9 (0.4%)
NA	1 (0.0%)
How would you describe your general health?	
Very good	527 (23.7%)
Good	1276 (57.3%)
Fair	394 (17.7%)
Poor	28 (1.2%)
Are you currently diagnosed with any of the following health conditions?	
Type 1 diabetes	8 (0.4%)
Type 2 diabetes	97 (4.4%)
Hepatic steatosis (fatty liver disease)	34 (1.5%)
Hypertension (high blood pressure)	417 (18.7%)
Hyperlipidemia (high cholesterol)	234 (10.5%)
COPD	19 (0.9%)
Heart disease	46 (2.1%)
Cancer	22 (1.0%)
Autoimmune	157 (7.1%)
Mental illness	241 (10.8%)
Physical disability	43 (1.9%)
Allergies	479 (21.5%)
Asthma	213 (9.6%)
Epilepsy	4 (0.2%)
Gastrointestinal issue	183 (8.2%)
Other	259 (11.6%)
None	890 (40.0%)
Do you take any prescribed medications?	
None	824 (37.0%)
Yes, 1 medication	503 (22.6%)
Yes, 2 medication	370 (16.6%)
Yes, 3 medication	230 (10.3%)
Yes, 4+ medication	298 (13.4%)
In the past 4 weeks I have been...	
Diagnosed with COVID-19	8 (0.4%)
Hospitalized	17 (0.8%)
Quarantined	55 (2.5%)
Self-isolating	363 (16.3%)
Under a shelter or stay at home order	198 (8.9%)
Social distancing	2148 (96.5%)

**Table 2:** Overall sociodemographic characteristics for all respondents (*n* = 2225).

	Meaningful Weight Loss Group (>=5%)	Moderate Weight Loss Group (2–5%)	Low Weight Loss Group (<2%)	Overall *p*-Value	Meaningful vs. Low *p*-Value	Meaningful vs. Moderate *p*-Value	Moderate vs. Low *p*-Value
	**n = 618**	**n = 236**	**n = 171**				
Mental health quality of life (1–5)	2.55 (1.1)	2.8 (1.15)	2.97 (1.17)	<0.001 *	<0.001 *	0.004 *	0.13
Depression frequency over the past 30 days				<0.001 *	<0.001 *	0.10	0.09
Not at all	163 (26.4%)	56 (23.7%)	26 (15.2%)				
Occasionally	393 (63.4%)	143 (60.6%)	108 (63.2%)				
More than half the days	48 (7.8%)	26 (11.0%)	22 (9.3%)				
Nearly every day	14 (2.3%)	11 (4.7%)	15 (6.4%)				
Anxiety frequency over the past 30 days				<0.001 *	0.0004 *	0.01	0.10
Not at all	86 (13.9%)	35 (14.8%)	12 (7.0%)				
Occasionally	393 (63.6%)	126 (53.4%)	96 (56.1%)				
More than half the days	105 (17.0%)	50 (21.2%)	44 (25.7%)				
Nearly every day	34 (5.5%)	25 (10.6%)	19 (11.1%)				
Work-life balance (1–10)	6.3 (2.16)	5.99 (2.23)	5.77 (2.15)	0.009 *	0.004 *	0.07	0.30
How would you rate your current sleep habits? (1–10)	5.95 (1.87)	5.94 (2.07)	5.69 (2.06)	0.29	--	--	--
Flourishing (8–56)	48.17 (5.93)	47.24 (6.47)	47.02 (6.42)	0.03 *	0.04	0.06	0.74

Note: For continuous variables, means are displayed, with standard deviations in parentheses. Mental health quality of life was reverse-scored with lower scores indicating higher mental health quality of life. *p*-values were obtained from ANOVAs. For categorical variables, Ns are displayed, with percentages in parentheses. *p*-values were obtained from chi-square tests. * *p* < 0.05.

**Table 3:** Psychosocial characteristics by engagement at weeks 5 and 17 (*n* = 2058).

	High Engagement Tertile	Medium Engagement Tertile	Low Engagement Tertile	*p*-Value	High vs. Low *p*-Value	High vs. Moderate *p*-Value	Moderate vs. Low *p*-Value
	**n = 686**	**n = 686**	**n = 686**				
Mental health quality of life (1–5)	2.52 (1.09)	2.64 (1.97)	5.66 (1.99)	<0.001	<0.001	0.04	0.002
Depression frequency over the past 30 days				<0.001	<0.001	0.05	<0.001
Not at all	209 (30.5%)	165 (24.1%)	138 (20.1%)				
Occasionally	415 (60.5%)	449 (65.5%)	414 (60.3%)				
More than half the days	47 (6.9%)	50 (7.3%)	95 (13.8%)				
Nearly every day	15 (2.2%)	22 (3.2%)	39 (5.7%)				
Anxiety frequency over the past 30 days				<0.001	<0.001	<0.001	<0.001
Not at all	83 (12.1%)	100 (14.6%)	78 (11.4%)				
Occasionally	456 (66.5%)	416 (60.6%)	393 (57.3%)				
More than half the days	112 (16.3%)	120 (17.5%)	140 (20.4%)				
Nearly every day	35 (5.1%)	50 (7.3%)	75 (10.9%)				
Work-life balance (1–10)	6.36 (2.13)	6.17 (2.25)	5.84 (2.24)	<0.001	<0.001	0.11	0.007
How would you rate your current sleep habits? (1–10)	6.13 (1.79)	5.93 (1.97)	5.66 (1.99)	<0.001	<0.001	0.05	0.01
Flourishing (8–56)	48.63 (5.73)	47.85 (5.69)	47.13 (6.39)	<0.001	<0.001	0.01	0.03

Note: High engagement tertile encompasses z-scores greater than or equal to 1.68 for week 5 participants or 1.93 for week 17 participants. Medium engagement tertile encompasses z-scores greater than −1.06 and less than 1.68 for week 5 participants or greater than −1.31 and less than 1.93 for week 17 participants. Low engagement tertile encompasses z-scores less than or equal to −1.06 for week 5 participants or −1.31 for week 17 participants.

**Table 4:** Backward stepwise regression model predicting weight loss at week 17 (*n* = 1025).

	β (95% CI)	*p*-Value
Children living at home		
None	--	--
1–2 children	0.99 (0.48 to 1.50)	<0.001 *
3–4 children	0.16 (−0.75 to 1.50)	0.73
5+ kids	−0.04 (−5.86 to 8.78)	0.99
Gender		
Female	--	--
Male	3.31 (2.64 to 3.97)	<0.001 *
Other	13.23 (5.53 to 20.94)	<0.001 *
Prefer not to answer	−0.33 (−4.46 to 3.81)	0.88
Anxiety frequency over the past 30 days		
Not at all	0.83 (−0.03 to 1.70)	0.06 +
Occasionally	0.94 (0.31 to 1.57)	0.004 *
More than half the days	--	--
Nearly every day	0.25 (−0.78 to 1.28)	0.63
Engagement tertile		
Low	−4.75 (−5.34 to −4.16)	<0.001 *
Medium	−1.45 (−2.05 to −0.87)	<0.001 *
High	--	--
Initial BMI	0.11 (0.08 to 0.14)	<0.001 *

Note. Only predictors that remained in the final model after backward stepwise elimination are displayed. * *p* < 0.05; + *p* < 0.10.

## Data Availability

Restrictions apply to the availability of these data. Data was obtained from Noom and are available by request from the corresponding author with the permission of Noom.
